# Homeostasis limits keratinocyte evolution

**DOI:** 10.1073/pnas.2006487119

**Published:** 2022-08-23

**Authors:** Ryan O. Schenck, Eunjung Kim, Rafael R. Bravo, Jeffrey West, Simon Leedham, Darryl Shibata, Alexander R. A. Anderson

**Affiliations:** ^a^Integrated Mathematical Oncology Department, H. Lee Moffitt Cancer Center and Research Institute, Tampa, FL 33612;; ^b^Wellcome Centre for Human Genetics, University of Oxford, Oxford OX3 7BN, United Kingdom;; ^c^Department of Pathology, Keck School of Medicine of the University of Southern California, Los Angeles, CA 90033

**Keywords:** somatic evolution, keratinocyte biology, mathematical modeling, carcinogenesis

## Abstract

Recent studies have revealed that normal human tissues accumulate many somatic mutations. In particular, human skin is riddled with mutations, with multiple subclones of variable sizes. Driver mutations are frequent and tend to have larger subclone sizes, suggesting selection. To begin to understand the histories encoded by these complex somatic mutations, we incorporated genomes into a simple agent-based skin-cell model whose prime directive is homeostasis. In this model, stem-cell survival is random and dependent on proximity to the basement membrane. This simple homeostatic skin model recapitulates the observed log-linear distributions of somatic mutations, where most mutations are found in increasingly smaller subclones that are typically lost with time. Hence, neutral mutations are “passengers” whose fates depend on the random survival of their stem cells, where a rarer larger subclone reflects the survival and spread of mutations acquired earlier in life. The model can also maintain homeostasis and accumulate more frequent and larger driver subclones if these mutations (NOTCH1 and TP53) confer relatively higher persistence in normal skin or during tissue damage (sunlight). Therefore, a relatively simple model of epithelial turnover indicates how observed passenger and driver somatic mutations could accumulate without violating the prime directive of homeostasis in normal human tissues.

## Significance

Human skin is riddled with mutations creating subclones of variable sizes. Some of these mutations are driver mutations, implicated in cancer development and progression, that appear to be under positive selection due to their relative sizes. We show how these driver and nondriver “passenger” mutations encode their history of division and loss within the tissue using a simple model combined with realistic mutation tracking. Using a three-dimensional in silico homeostatic epidermis model, we reveal that many mutations likely lack functional heterogeneity and are, instead, simply those that arise earlier in life within the basal layer. We use our model to reveal how functional differences conveyed by driver mutations could lead to a persistence phenotype while maintaining homeostasis.

Recent studies have documented that substantial numbers of mutations accumulate in normal human tissues, with evidence for selection of canonical, oncogenic driver mutations (1–6). Typically, selection for these driver mutations is modeled by increased cell proliferation; however, requirements of skin homeostasis impose spatial constraints that inherently limit selective sweeps. Because these mutations do not appear to disrupt homeostasis, these mutations can also provide important new information on normal human tissue dynamics, which are typically studied with experimental fate markers in animal systems.

Although substantial numbers of mutations accumulate with age, and a quarter of all cells carry driver mutations, skin thickness and mitotic activity do not significantly change. A controversy currently exists between our understanding of mutation selection and neutral evolution within tissues (1), in part because our understanding of how a mutant clone is able to expand within normal tissue is completely lacking. Furthermore, most mutations are passengers and skin subclone size/frequency distributions are consistent with neutral evolution or the absence of detectable selection. There is, however, strong evidence of selection by driver mutations (NOTCH and TP53) manifested by dN/dS ratios and their generally larger subclone sizes in normal skin (1). Varying exposures to sunlight between individuals also complicate our understanding of mutation accumulation. Studies modeling the epidermis and clone sizes thus far explore whether the methodologies available are capable of accurately measuring neutrality in normal tissue through nonspatial or simple, nonhomeostatic two-dimensional mechanistic models (7, 8). Alternatively, more complex models that attempt to capture homeostasis are uninterested in the underlying mutation dynamics (9). The challenge is to develop a model that integrates selection, neutral evolution, and sunlight. The reward would be a realistic model of lifelong human skin-cell dynamics.

## Modeling Approach and Keratinocyte Behavior

In the scope of the epidermis, first principles dictate a constant cell number through equal loss and replacement of cells, a constant tissue height, and constant stem cell numbers. Using these principles, we built a three-dimensional hybrid cellular automaton (3D-HCA) using the hybrid automata library (HAL) (10), a comprehensive cell-based modeling framework allowing users to focus on the development of model specific functionalities. This 3D-HCA allows us to mechanistically model the human epidermis. We based our model on a simplified version of the hybrid multiscale mathematical model of normal skin (vSkin) we previously developed (9). This model examined homeostasis across the dermis and epidermis to investigate the role of senescent fibroblasts in melanoma initiation and progression. We use our simplified model to investigate the clonal dynamics of keratinocyte populations (*SI Appendix*, Fig. S1). While we model keratinocyte dynamics, we do not explicitly consider melanocytes and stromal cells (i.e., cells not found in the epidermis), but rather we use a single diffusible growth factor to represent the role of fibroblasts in defining the stem niche within the basal layer. We constrain our simulation area based on the different-sized patient biopsies we will compare with our results (0.75,  1.0, and $3.14\;{\text{mm}}^{2})$, assuming a median cell size of approximately 15 µm, providing a 3D tissue of appropriate cell numbers within a given area.

The growth factor provides a mechanistic driver governing keratinocyte apoptosis and proliferation, defined by a partial differential equation that dictates the diffusion rate, consumption rate by keratinocytes, and decay rate throughout the simulated domain over time. We assume a constant source of growth factor along the basal layer, mimicking a fibroblast source. The growth factor rapidly sets up a gradient throughout the domain that governs keratinocyte cell behavior, combined with the cell interactions, resulting in an emergent homeostatic epidermis (the full mathematical description of diffusion and boundary conditions can be found in *SI Appendix*).

**Fig. 1.**
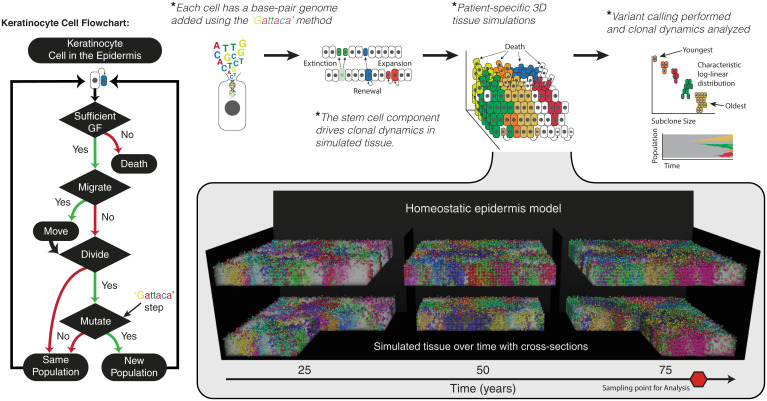
Homeostatic epidermis model with high-resolution genomes. The homeostatic epidermis is composed of individual keratinocyte cells that are governed by a set of decisions (flowchart). Spatial structure of the 3D model and a growth factor (GF) diffusible gradient governs the loss/replacement in the stem-cell niche at the basal layer. The model is entangled with DNA sequence data by the Gattaca step, where base-pair mutations can be introduced with every in silico cell division. These mutations act as cell-fate markers and different subclones are represented by different cell colors. Therefore, cells in the simulated skin produce a patchwork of different-sized subclones, where each colored population differs by at least one mutation.

Keratinocytes undergo both death and division based on the underlying growth factor concentration. In our model, stem cell behavior is driven by the growth factor resulting in a niche driven phenomenon where the bulk of division occurs within the basal layer. Importantly, when a cell undergoes division, this is the only time that it can displace neighboring cells. As a keratinocyte divides, a parameter governs where the daughter cell is located such that it can displace one of its four neighbors or be placed directly above the cell undergoing division. When a cell occupies the space where a daughter cell will occupy, the cell occupying that space is moved up one lattice position, with all cells in the way following the same fate of upward movement. This knock-on effect facilitates balanced turnover in the epidermis and provides a mechanism to exploit when introducing nonfunctional heterogeneity. As these keratinocytes move toward the surface of the epidermis, they are subjected to an ever-increasing chance of death due to the decreasing concentration of growth factor. Together, this dynamic homeostatic epidermis model with balanced birth/death recapitulates the appropriate cell age, density, and turnover (Fig. 1 and *SI Appendix*, Fig. S1) of real epidermis.

Niche-driven behavior, while simplified, is an appropriate reduction in a homeostatic epidermis model. Several studies have provided insights into the heterogeneity of stem-cell populations that influence clonal dynamics of the epidermis (11, 12). The primary types of stem cells sustaining the epidermis are holoclones, undergoing extensive self-renewal. The remaining stem cells, transiently dividing before being lost, meroclones and paraclones make up the bulk of the cells of the epidermis. We can see that the observed frequency of holoclones within the population is a rare event (those that persist within the basal layer the longest periods of time; *SI Appendix*, Fig. S1). Most recently, in a commendable study by Hirsch et al. we see that the proportion of holoclones is between 1.92 and 11.26%; we observe that 95% of stem cells within the basal layer are lost within 4 mo, consistent with the findings that meroclones and paraclone half-lives are 3 to 4 mo (11). The resulting distribution consists of predominately small clones as most clones are lost regularly (nonholoclones) with few persisting in the basal layer where the overall half-life of all stem cells is ∼4 wk, an observation invariant to differing domain sizes (*SI Appendix*, Fig. S1*C*).

Within our homeostatic epidermis approach, instead of a simple stochastic birth/death model in two dimensions or nonspatial approach we can see that wounded tissue regenerates and heals due to the model’s capability of regenerating tissue in response to a surge of our growth factor. This growth factor increases in concentration in the wounded space where consumption of the diffusible is no longer taking place by the lost keratinocytes. We use experimentally derived measurements showing a progenitor loss/replacement rate of 0.51 per week (7) and calculate a basal layer loss/replacement rate that we then use to parameterize the model (basal/progenitor layer half-life of ∼30 d) (*SI Appendix*, Fig. S1*E*). While this niche-driven behavior allows for some transient amplifier cell division above the basal layer these cells are inconsequential as they are lost in the stratum cornea (*SI Appendix*, Fig. S1). Overall population size (mean cell number of ≈120,000 within a simulated 1-mm^2^ biopsy) is maintained by balanced birth with death, where the top layers of the epidermis transition into the stratum cornea due to low growth-factor concentrations. Hence, random stem-cell survival and homeostasis results in a mosaic of subclones of different sizes and ages.

### Incorporation of In Silico Genomes.

To incorporate patient mutational data, we developed the “Gattaca method” in which 72 specific genes, tracked at base-pair resolution with gene-specific mutation rates, are imbedded within each keratinocyte, accurately capturing the mutation distribution consistent with ultraviolet (UV) exposure (13). “Gattaca” stands for the four DNA bases, GATC, and reflects that individual base changes are used as intrinsic cell fate markers that can be interrogated by DNA sequencing. Each cell contains a base-pair resolution of 72 genes with genomic positional information constructed from the reference hg19 genome using ANNOVAR (14). Each gene has a gene-specific mutation rate such that the mean mutation rate ($\mu_{g})$ is normalized to $\approx 3.2\times 10^{-9}\;{\text{bp}}^{-1}{\text{division}}^{-1}$ [likely a conservative estimate, but normal somatic mutation rates can vary wildly (15–18)]. This method allows for a more realistic mutation accrual, since a genome-wide mutation rate would be biologically unrealistic due to a large number of covariate factors (e.g., level of expression, location on chromosome, and GC content, etc.) leading to differences in mutation rates (19). Specifically, a mutation rate applied uniformly across the genome leads to the longest genes, such as ERBB4, PTPRT, and BAI3, accumulating the most mutations (in our model) while NOTCH1-4 would acquire almost no mutations. This is not observed in normal epidermis, where NOTCH1 and a number of various other shorter genes are frequently mutated.

Upon initialization of the model we construct Poisson distributions for each gene given the mutation rate, $\mu_{g}$, and the length of the gene, $L_{g}$, providing the expected number of mutations upon division. Where the number of mutations for each gene, $X_{g}$, is $X_{g}∼\text{Poisson}\left(\mu_{g}\times L_{g}\right),\;$ and the specific base affected is determined by a multinomial distribution constructed for each A, C, T, and G, allowing us to define and capture the specific mutational signature observed in the data. Keratinocyte (and melanocytes not considered here) contain disproportionately more C > T transitions (1, 3); this needs to be accounted for by adjusting the probability of a C > T mutation occurring versus all other mutation types. Here we set the probability of a cytosine mutation to 0.5 and split the remaining bases to an equal probability. Finally, we determine the position within the gene by applying a uniform probability to all of the same bases within the gene. All ancestral mutations are inherited by daughter cells. This method, adapted from the Gattaca framework and incorporated into each cell, allows us to fully represent the tissue architecture of a homeostatic epidermis with realistic mutation accumulation (Fig. 1 and Movie S1).

### Comparing Neutral Simulations to Patient Data.

**Fig. 2.**
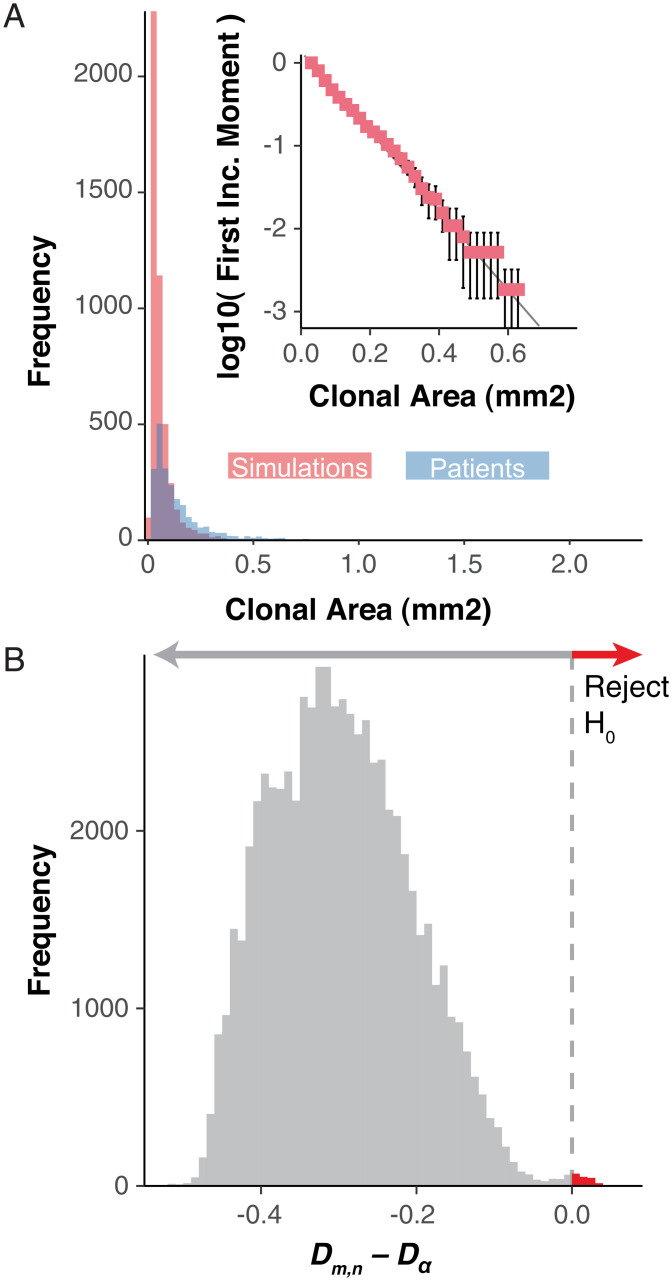
Homeostasis imposes log-linear clonal distributions. Neutral model dynamics from 3D simulations of various sizes where *A* is the cumulative clonal area frequencies of all patient biopsies (blue) and randomly chosen simulations for each of the patient-specific biopsies such that the number of simulations chosen is equal across the three simulated sizes and the sum of the number of biopsies equals that of the total biopsies for the patient (red). (*Inset*) The log-10 transformed first incomplete moment for the same random sampling of patient comparable simulations for PD20399 (error bars denote SD for 100 repeated samplings). (*B*) The difference from the Komlogrov–Smirnov test statistic (D_m,n_) and critical value (D_α_) for all patient biopsies to patient-specific model simulation’s first incomplete moment distributions for all simulation sizes. The red arrow denotes comparisons where the null hypothesis can be rejected.

**Fig. 3.**
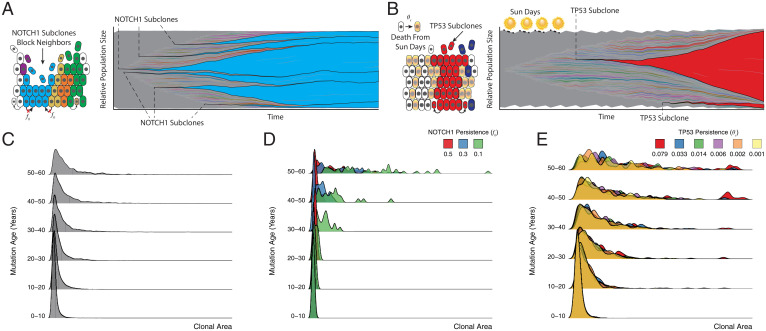
Homeostasis, a priori, constrains clones in a functionally heterogeneous tissue, dictating an age-dependent clonal expansion. Clonal dynamics in homeostasis are a function of complex interactions between the microenvironment and the external environment. During homeostasis, every cell is equal relative to its neighbors, i.e., neutral. NOTCH1 disrupts neutral dynamics by offering a slight advantage via a blocking probability ($f_{0}$) which prevents neighboring cells from dividing into their positions (*A*), whereas TP53 mutations are not subject to UV damage, when a proportion (θs) of non-TP53 mutant cells may be killed by UV damage (*B*). (*A* and *B*) Next to each schematic are shown clonal expansions for a single simulation for NOTCH1-advantaged clones, blue, and TP53-advantaged clones, red, in a 1-mm^2^ simulation to 58 y, respectively. The age dependence is shown in the density plots broken by mutation age for 100 replicate simulations up to 58 y for neutral (*C*), NOTCH1, with varying strengths ($f_{0}$) (*D*), and TP53, with varying strengths (θs) (*E*).

Simulations of variable biopsy sizes (0.75,  1.0, and $3.14\;{\text{mm}}^{2})$, age-matched to comparable patients, reconstructs a log-linear subclone size–age distribution with neutral mutations that easily captures the extent of clone size variability within patients (Fig. 2 *A* and *B*). We see that small clones dominate the highest frequencies, while a heavy right-tailed distribution is indicative of the clonal exponential size dependency (Fig. 2 *A* and *B*) consistent with patient data (3, 7, 8). We then performed pairwise Kolmogrov–Smirnov tests for each patient and the simulated biopsy’s first incomplete moment distribution. We fail to reject the null hypothesis that the distribution of clone sizes are from different distributions, for the vast majority of our simulation versus patient biopsy comparisons, but are able to reject the null for a select few comparisons (Fig. 2*B* and *SI Appendix*, Figs. S2–S5). However, for all patient biopsies at least one of the patient specific simulations fails to reject the null hypothesis. In a homeostatic epidermis, most subclones rife with nonfunctional (i.e., neutral) mutations are small, recent, and destined for extinction due to random stem-cell turnover (Figs. 2 *A* and *B* and 3*E* and *SI Appendix*, Fig. S6). Persistent, older subclones are rarer and larger (Fig. 3*D*). Hence, the observed log-linear subclone mutation size distributions (Fig. 2*B*) are an emergent property of the homeostatic constraints of normal human skin. These results reveal that much of the clinically observed mutation data largely reflect stochastic stem-cell survival, where large subclones (e.g., greater mutation frequencies) represent the survival and expansion of older lineages.

### Modeling Fitness Advantage in NOTCH1 Mutant Keratinocytes.

**Fig. 4.**
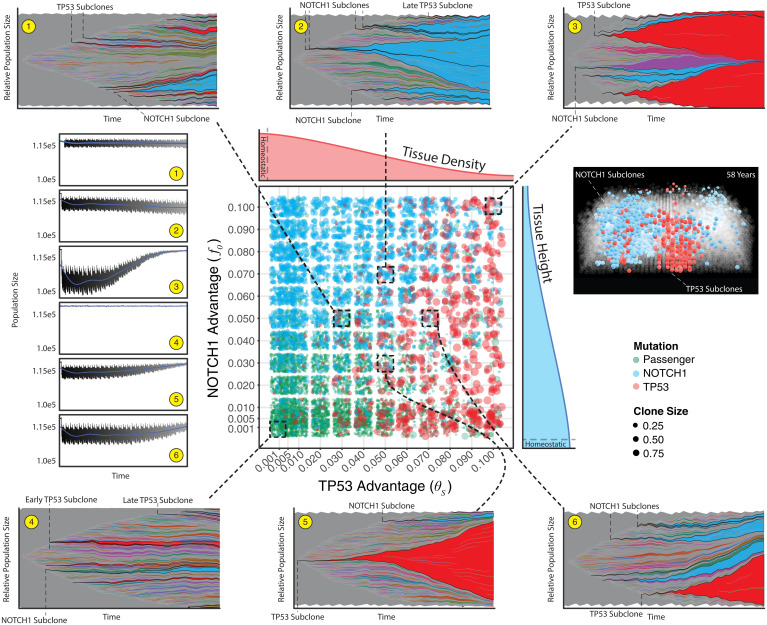
Combined effects reveal homeostatic recovery and recapitulate patient clone distributions. The primary, central plot reveals clone sizes for 1-mm^2^ simulations for four different ages with five replicates across a manually defined scale of parameter pairs for NOTCH1 (f0) and TP53 (θs) advantages (*n* = 2,880 simulations). Density subplots show an idealized example of how each mutation type effects homeostasis (red and blue for TP53 and NOTCH1, respectively). The dashed boxes and lines show the evolutionary dynamics for a single simulation for a 58-y simulation (population frequencies greater than 0.001) for six parameter pairs and the corresponding overall population sizes over time (1–6 yellow circles, blue line is a locally weighted smoothing line). For a single parameter pair (θs = 0.03, f0 = 0.05) a 0.4-mm^2^ tissue is shown where only NOTCH1 (blue) and TP53 (red) clones are colored.

**Fig. 5.**
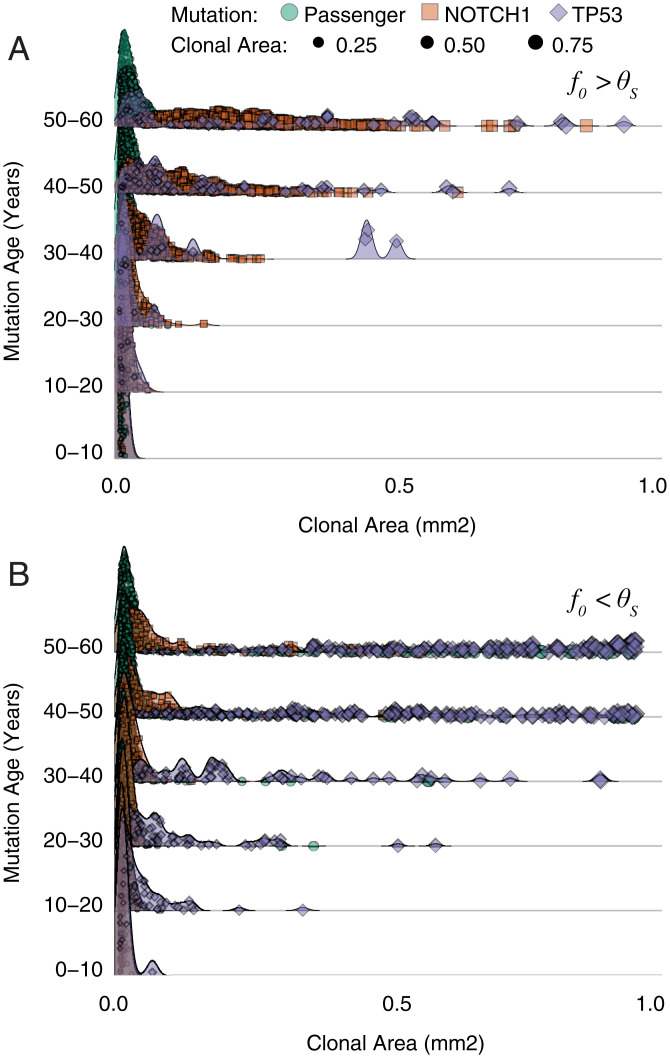
Combined effects adhere to age-dependent expansions. All 58-y 1-mm^2^ paired parameter simulations from Fig. 3 are displayed where TP53 persistence (θs) is greater than NOTCH1 persistence (f0) (*A*) and f0 > θs (*B*) (for f0 = θs see *SI Appendix*). Individual clones are broken into three groups (passenger, NOTCH1, and TP53) with corresponding sizes of each clone.

Previously, NOTCH1 and TP53 mutations were observed to have larger subclone sizes relative to neutral mutations (1), indicating positive selection. Interestingly, age-related clonal size dependencies also exist even for advantageous driver mutations in the homeostatic simulations (Fig. 3 *D* and *E*). Unlike Lynch et al. (3) which lacks the homeostatic control in a two-dimensional model of the epidermis, our findings implicate the constraints of the homeostatic tissue architecture as a primary factor in defining the age-related exponential size dependency of selective mutations and their subclones. To better define this selection within the context of homeostasis and without changing division rates directly, we simulated a decreased ability of NOTCH1 mutant subclones to be displaced by neighboring cells, as observed experimentally (12). Mechanistically, we introduce functional change to the model when a NOTCH1 mutation occurs through a parameter (blocking probability, $f_{o}$) leading to increased persistence of NOTCH1 mutant cells over dividing nonmutant cells (*SI Appendix*, *NOTCH1 Advantage*). This allows a NOTCH1 mutant to persist in the basal layer longer than a nonmutant cell. The increased persistence of NOTCH1 mutated subclones increases both subclone sizes and frequencies, consistent with positive selection (Fig. 3*D*). However, the requirement to maintain homeostasis indicates this mechanism comes with a cost, which inherently limits the strength of selection (Figs. 3*A* and 4). The stronger the advantage, the greater the loss of homeostasis, creating a catch-22 where a NOTCH1 mutant clone with too much of a selective advantage destroys the epidermis, which is not observed in mice or human epidermis. Therefore, the simulations indicate a “homeostatic” limit to the strength of NOTCH1 selection such that young, rapidly expanding large NOTCH1 subclones are seldom observed (Figs. 3*E* and 5 *A* and *B*). This is consistent with the observation that patient NOTCH1 mutations are smaller but more frequent than TP53 mutations (1, 3). Instead, similar to the neutral mutations, the largest NOTCH1 subclones are the oldest NOTCH1 mutations. Although driver mutations can only confer limited selective advantages without disrupting homeostasis, even small persistence advantages expressed at earlier patient ages pays dividends and inherently leads to larger and more frequent NOTCH1 subclones in adult skin (relative to neutral mutations).

### Modeling TP53-Mediated Advantage in the UV-Exposed Epidermis.

TP53 mutations protect against cell death and sunlight enhances selection because TP53 subclones are smaller in non-sun-exposed skin (20). Normal sun-exposed skin does not exist because the extent of sun exposure varies massively between patients. Here we incorporate this important environmental variability through “sun days,” where our homeostatic tissue takes a sun-filled vacation and a proportion of non-TP53 mutant cells are killed by UV irradiation (Fig. 3*B*). Introducing functional heterogeneity by a TP53 advantaged clone does not involve a cell-intrinsic parameter. Rather, UV damage kills cells on a given sun damage day, S, where the proportion of non-TP53 mutant cells killed is given by $\theta_{s}$ (Fig. 3*B*). The TP53 mutant cells are subject to the same dynamics aside from this reprieve from UV-induced death. We determined the parameters to use for the sun damage day set, defined by the number of days with UV exposure and the spacing of those days throughout the year, and $\theta_{s}$ by approximating our 3D-HCA using an ordinary differential equation (ODE) that models keratinocyte population dynamics in a space-limited condition as a simple logistic growth equation (*SI Appendix*, Eq. **9**). This ODE approximation allows us to assess the full growth dynamics of the epidermis with UV exposure over a vast parameter space. This would be computationally unfeasible for the full parameter space using the 3D-HCA. However, using the ODE we can then assess parameters of interest and subsequently explore the clonal dynamics in the full 3D-HCA. Due to sun exposures varying within the lifetime of each individual we assume that a baseline UV exposure is taken into account for the parameterization of the model, encompassing the empirically derived turnover (birth/death) of cells within the basal layer. Thus, it is necessary to have a method of UV damage and subsequent TP53 mutant advantaged clones without a direct increase in proliferation rates. The number of sun damage days within a given year defines the sun day set (*S*) and can take on a number of values depending on how those sun damage days are spaced throughout the year. There are 365 possible sun damage days a year. It is unlikely that an individual would be subjected to all of these sun days, but there is a high degree of variability between individuals. This ODE approximation takes a relatively complex model and reduces its complexity and the spatial dynamics necessary to evaluate clonal dynamics in order to assess UV damage on the spatial, homeostatic model (*SI Appendix*, Figs. S3 and S4; additional theory in *SI Appendix*).

In our model, a TP53 mutation provides no inherent fitness advantage *ipso facto*. Its ultimate fitness advantage is realized upon UV exposure where nonmutant neighbors may be killed, allowing the clone to expand into these now-empty spaces (Fig. 3*E*). Thus, depending on the combined UV exposure (based on the number of sun days and their frequency) a TP53 mutant clone can take an appreciable time to expand within the tissue (Fig. 3*B*). If the UV exposure stops, the clone is subject to the same mechanisms of division and its only advantage may be its size. Early on, this advantage is a powerful one, but the overall clonal dynamics will return to neutral drift. Furthermore, this same TP53 mechanism can prevent the spread of future TP53 clones that arise later. These later-arising TP53 mutant clones are unable to realize their fitness advantage if their neighbors are already TP53-mutated, reinforcing the clonal size dependency where the oldest clones are the largest clones even within the TP53 mutant subpopulations (Fig. 3*E*). This mechanism, unlike NOTCH1, does not intrinsically increase persistence but protects TP53 subclones from death after sun exposure, which allows rapid expansion as normal cells are eliminated when skin is exposed to more sun days (Fig. 3*E*), consistent with mice studies that show short-term expansions of TP53 mutant clones upon UV damage (20). However, similar to NOTCH1, simulated TP53 subclonal expansions are also constrained by homeostasis, because too much simulated UV exposure causes a loss in tissue density and homeostasis.

Given the patient data (and normal skin in general) is most likely from patients with significant sun exposure, we consider the situation where both NOTCH1 and TP53 are found within the same simulated biopsy (Figs. 4 and 5). In the absence of sunlight, TP53 mutations are subject to drift, along with all other mutations. Consistent with patient data we see evidence of selection with more frequent occurrences of NOTCH1 mutations at subclone sizes larger than neutral mutations. Upon sun exposure, TP53 mutations are able to expand, even when they arise later in life, thereby leading to larger TP53 mutant subclones and shifting the age-dependent distribution to a more recent point in time. Passenger mutations are occasionally carried to larger clonal areas, but they are mainly relegated to the lowest frequencies (Fig. 4). Interestingly, sunlight can reduce the selective effects of NOTCH1 mutations (i.e., reduce subclone sizes) because TP53 mutant subclones expand with UV irradiation (Fig. 4). Even with the presence of sunlight and both mutations, we reveal an age-dependent log-linear relationship consistent with patient data where the earliest mutations expand to appreciable sizes within the tissue (Fig. 5).

## Discussion

Here we have presented a data-driven in silico model able to capture the homeostatic cell dynamics of sun-exposed human epidermis with high-resolution base-pair tracking using the “Gattaca” method embedded within the HAL (10) framework. Our in silico epidermal model reveals that mutational persistence is the key to observed subclone frequency–size distributions. Strikingly, both neutral and driver mutations exhibit similar frequency–size distributions broadly consistent with neutral evolution, reflecting that random stem-cell turnover is a salient feature of normal human epithelial homeostasis. Although mitotic stem cells accumulate mutations, random stem-cell death with replacement typically leads to mutation flushing such that most de novo mutations are lost or are limited to small subclones. This work broadens our current understanding of selection and fitness acting in a homeostatic, normal tissue, where subclone size more reflects persistence rather than selective sweeps, with larger subclones being predominately older subclones. Although the underlying skin model does not include all the biological variables underlying skin integrity, it illustrates that by explicitly considering the prime directive of normal tissue to maintain homeostasis as well as the effects of environmental damage we can readily account for the major features of patient normal skin mutation data.

We show that NOTCH1 and TP53 mutations (possibly other oncogenic mutations as well) must arise early to expand to observed clonal frequencies in patients. We also know that various nonmelanoma skin cancer incidences increase with age after a lifetime of sun exposure. Together, this indicates that mutations, while a necessary precursor, are insufficient to be the sole drivers in the transition to cancer. This also supports the idea that the most important time to guard tissue from UV damage is generally in the first half of one’s life. This is a challenging conclusion to reach based on data alone as the timing of mutations can be difficult to resolve in normal human tissue. The homeostatic epidermis model reveals that it may be possible to infer sun exposure over a lifetime by examining clone sizes, mutation types, and spectra. The power of this approach would be determined by the resolution of the longitudinal dataset that would have the necessary information and the statistical inference methodology that links our 3D-HCA model with that data. The model presented here assumes equal UV exposures repeated yearly to reduce the complexities of human sun exposure over one’s lifetime. However, it is more likely a combination of mutation induction timing (e.g., TP53) along with the timing of the change in selective forces (e.g., irregular UV exposure) combined with any additional fitness advantages/disadvantages the cell may have. Further experimentation and modeling would be needed to evaluate this fully.

We do not claim that the 3D-HCA model accurately captures stem-cell dynamics, which have complex biological mechanisms; rather, this model strives to provide a means to explore mechanisms of increased fitness in normal, homeostatic tissue. This provides a simple homeostatic framework for future researchers to model their hypothesized mechanisms within squamous tissue to better inform patient data collection and measurements as well as refine in vivo and in vitro modeling approaches tailored to their hypothesis. Complimentary to the findings here, there is supportive evidence of homeostasis spatially constraining clones that have a fitness advantage in the normal esophagus (21), another squamous cell epithelial tissue that has mutation accumulation similar to that of normal skin (NOTCH1 and TP53). The esophagus lacks UV exposure but is subject to regular chemical and thermal insult where similar fitness advantages for NOTCH1 and TP53 presented here could be conceivable. Ultimately, the increasing availability of mutation data from normal human tissues combined with new advances in lineage clone tracing in vivo will further aid to parameterize our agent-based tissue simulations, as well as many others, for future experimental validation and hypothesis testing.

## Materials and Methods

### Patient Data.

Patient data were previously procured and exhaustively characterized (1). However, the data are filtered to remove mutations present in multiple biopsies as this may prove problematic in its interpretation, as previously noted (7). The clonal area is taken as the product of the biopsy size (square millimeters) and twice the variant allele frequency. This curated dataset provides the necessary information to perform direct comparisons with simulation data.

### Model Data.

Once a simulation is complete the true variant allele frequency ($VAF_{t}$) is calculated as the quotient of the total number of cells carrying that mutation over the product of twice the total population size. This neglects copy number variation as this is not tracked within the model. However, high-throughput sequencing methodologies rarely yield the true variant allele frequency and result in imperfect information, such as that collected here. We employ and build upon a method to simulate sequence data yielding a simulated variant allele frequency ($VAF_{s}$) (22). The authors determine $VAF_{s}$ by drawing the number of variant reads, $f_{i}$ for any given mutation *i* from a binomial distribution where the success probability is given by $VAF_{t}$ and the number of trials is itself binomially distributed and yields the total number of reads ($D_{i}$):

$$\begin{array}{c} f_{i}∼B_{o}\left(n=D_{i},p=VAF_{t}\right) \end{array}.$$

However, here $f_{i}$ utilizes a higher-resolution approach given the higher-quality sequence data that are used for comparisons. Martincorena et al. (1) went to great lengths to generate high-resolution data by sequencing up to an average depth of 1,000× (patient PD13634; while all others are ∼500×) to ensure the capture of low-frequency variants, approaching the error rate of the sequencing method used. In order to incorporate the error and variance around the depths at each variant site that would be introduced by the sequencing methods we define $D_{i}$ by drawing from a gamma distribution. The gamma distribution shape parameters, $k_{p}$ and $\theta_{p}$, are specific to each patient and determined by fitting each patient’s variant depth distributions using a maximum likelihood estimation [conducted in R (23) using the MASS package (24)]. $D_{i}$, whose parameters are specific for each patient, for each variant becomes

$$\begin{array}{c} D_{i}∼\mathrm{Gamma}\left(n=1,\;k=k_{p},\;\theta =\theta_{p}\right). \end{array}$$

Thus, as described above, the final frequency of a variant for comparisons is given as

$$\begin{array}{c} VAF_{s}=\frac{f_{i}}{D_{i}}. \end{array}$$

### Assessing Neutrality.

Most recently introduced mutations dominate the lowest frequencies and thus are difficult to observe. By using a variant allele frequency (VAF) cutoff of 0.005, the lowest VAF observed in the patient data, a large fraction of mutations is filtered out and analysis is conducted on what is likely to be observable within high-resolution patient sequence data.

Given a neutrally expanding keratinocyte population driven by progenitor proliferation with a basal progenitor loss/replacement rate given by rλ, it follows that the clone, n, distribution can be calculated for any given patient of age *t*:

$$\begin{array}{c} P_{n>0}\left(t\right)=\;\frac{1}{\ln\left(r\lambda t\right)}\frac{e^{-\frac{n}{r\lambda t}}}{n}. \end{array}$$

The cumulative probabilities of clone size distributions can then be used to assess neutrality, under an expectation of exponential size dependency given by the first incomplete moment:

$$\begin{array}{c} \mu_{1}\left(n,t\right)=\;\frac{1}{n\left(t\right)}\overset{N}{\underset{m=n}{\sum }}mP_{m}\left(t\right). \end{array}$$

Here, N represents the largest clone observed and 〈n(t)〉 is the average mutant clone size. This assessment of neutrality allows for a straightforward method when applied to simulation data and patient biopsies. Clone size can be calculated by 2*VAF*B, where B is either patient biopsy size or simulated biopsy area in square millimeters.

### Departure from Homeostasis.

In order to assess how the 3D-HCA behaves in response to functional heterogeneity being introduced through NOTCH1 and TP53 mutants we use a distance metric $d_{H}$. This distance metric summarizes the departure from homeostasis given a normal morphology such that the overall population size is constant throughout the simulation (e.g., all neutral simulations). It is defined as

$$\begin{array}{c} d_{H}=\log\left(\frac{1}{n}\;\sqrt{\overset{n}{\underset{i=0}{\sum }}{\left(N_{k}\left(t_{i}\right)-N_{s}\left(t_{i}\right)\right)}^{2}}\right), \end{array}$$

where $N_{k}$ and $N_{s}$ are the population size at the given time, $t_{i}$, for the homeostatic and simulation in question, respectively. This provides a single, relative value to assess how far from homeostasis any simulation departs.

## Supplementary Material

Supplementary File

Supplementary File

## Data Availability

Previously published data were used for this work (1). The model was created using the HAL framework (10) with a custom module (HAL framework available here: https://github.com/MathOnco/HAL) (25, 26). All source code specifically for this epidermis model is available on GitHub at https://github.com/MathOnco/HomeostaticEpidermis (27).
