# Supplementary material for: Homeostasis limits keratinocyte evolution
Source: Proc Natl Acad Sci U S A. 2022 Aug 23;119(35):e2006487119. doi: 10.1073/pnas.2006487119 (PMC9436311; doi:10.1073/pnas.2006487119)
Supplement: Supplementary File [file pnas.2006487119.sapp.pdf]

Classification: Biological Sciences; Evolution

Supplemental Text and Figures

Homeostasis Limits Keratinocyte Evolution

Ryan O. Schenck[1,2], Eunjung Kim[1], Rafael R. Bravo[1], Jeffrey West[1], Simon Leedham[2], Darryl Shibata[3,†], and Alexander R.A. Anderson[1,†,*]

Affiliations:

[1] Integrated Mathematical Oncology Department, H. Lee Moffitt Cancer Center and Research Institute, Tampa, FL 33612, USA

[2] Wellcome Centre for Human Genetics, University of Oxford, Oxford, OX37BN, UK

[3] Department of Pathology, Keck School of Medicine of the University of Southern California, Los Angeles, California 90033, USA

* Indicates the author to whom correspondence and material requests should be addressed.

† These authors share senior authorship.

Corresponding Author Contact: Alexander.Anderson@moffitt.org

Keywords: Somatic Evolution, Keratinocyte Biology, Mathematical Modelling, Carcinogenesis

Diffusion of the growth factor

The single microenvironmental factor governing keratinocyte apoptosis and proliferation is defined by a partial differential equation that dictates how our diffusible behaves throughout the system over time. Similar to Kim *et al.* (1) & Basanta *et al.* (2), due to the interactions between discrete, on lattice cells, and the continuous growth factor variable we describe the discretized version of the equation. Over time our growth factor diffuses from an imposed, unchanging source (E_C, Table S1) along the basal layer with a diffusion rate (ψ, Table S1), representative of fibroblast production from the dermis, is consumed by keratinocytes (K) at a consumption rate (γ, Table S1), and decays naturally (λ, Table S1). The spatial and temporal dynamics of this microenvironmental factor in its discretized form, using a first order Euler method, on a three-dimensional lattice is defined as the following difference equation:

$$\frac{[E(\eta, t+\delta t)] - [E(\eta, t)]}{\delta t} = \psi \Delta_3[E(\eta, t)] - \gamma[E(\eta, t)]K_\eta - \lambda[E(\eta, t)], \tag{1}$$

where $\eta \equiv (\eta_x, \eta_y, \eta_z)$, δt denotes the time step, K_η is set to 1 if the lattice point η is occupied by a keratinocyte and 0 otherwise. The Δ_3 denotes the central difference approximation of the Laplacian operator in three-dimensional space:

$$\Delta_3 f(\eta_x, \eta_y, \eta_z) \equiv \frac{f(\eta_x + h, \eta_y, \eta_z) + f(\eta_x - h, \eta_y, \eta_z)}{h^2} + \frac{f(\eta_x, \eta_y + h, \eta_z) + f(\eta_x, \eta_y - h, \eta_z)}{h^2} + \frac{f(\eta_x, \eta_y, \eta_z + h) + f(\eta_x, \eta_y \eta_z - h)}{h^2} - \frac{6 f(\eta_x, \eta_y, \eta_z)}{h^2} \tag{2}$$

where h is the grid size. Boundary conditions on the top ($\eta_z = Z, Z$: height) are no-flux $\left(\frac{\partial E}{\partial z}\Big|_{z=Z} = 0\right)$ and on the bottom ($\eta_z = 0$) are Dirichlet ($E(\eta_x, \eta_y, 0) = E_C$), while periodic boundary conditions are present on the left & right and front & back ($E(0, \eta_y, \eta_z)$= $E(X, \eta_y, \eta_z)$ & $E(\eta_x, 0, \eta_z)$= $E(\eta_x, Y, \eta_z)$). Each time step we solve equation (1) until steady state reached (<100 iterations). A steady state value of the growth factor is used to determine keratinocyte birth and death in the model.

Keratinocyte dynamics model

Cell death can occur in one of two ways at each time step for each cell (cell time step is equal to 1 day), either through apoptosis or random turnover. First, the cell is dependent upon growth factor concentration to prevent a cell from undergoing apoptosis. The level at which apoptosis can occur by chance (α, Table S1), is a model specific parameter and translates to a cell undergoing apoptosis when a randomly selected number $c_\alpha \in U(0,1)$ is less than $P_{Death\_E}(K_\eta) = \left(1 - \frac{E(\eta,t)}{\alpha}\right)^5$, (i.e., if $c_\alpha < \left(1 - [E]_\eta * \frac{1}{\alpha}\right)^5$, the keratinocyte K_η at a lattice point η dies). The exponentiation to the fifth power provides the appropriate distribution of keratinocyte deaths to prevent a completely flat epidermis surface and one that is highly irregular. While slightly arbitrary, five was chosen to balance an almost completely flat surface of the epidermis ($\rho > 5$) and one that is thicker with a lower density of keratinocytes due to the probability of death being more even across the concentrations of our growth (ρ=1). If a cell survives

in low growth factor concentrations or is not in an area with sufficiently low growth factor it is then subject to random death representative of an intrinsic probability of death ($P_{Death\_i}\,(K_\eta) = \theta$) where death will occur if a randomly selected number from (0,1), c_θ ,is less than θ (i.e., if $c_\theta < \theta$, $c_\theta \in U(0,1)$, then death occurs).

Provided a cell does not die within a given time step it is capable of moving to an empty lattice position in its 6 immediate nearest neighbors i.e. the von Neumann neighborhood ($N_{(x,y,z)}{}^v = \{(x,y,z+h),(x,y,z-h),(x+h,y,z),(x-h,y,z),(x,y-h,z),(x,y+h,z)\}$) . This location is determined by assessing if any empty lattice positions exist within the cell's neighborhood. If multiple spaces are empty, one is chosen randomly. The model specific parameter dictating the probability of movement ($P_{Move}(K_\eta) = \rho$, Table S1) governs the cells ability to move into that empty space so that when a randomly selected number c_ρ from (0,1) is greater than ρ, then the cell moves into that empty position (i.e., if $c_\rho \in U(0,1) \geq \rho$, the cell will move into a randomly selected empty neighborhood, $N_{(x,y,z)}{}^v$).

Cells divide based on the underlying growth factor concentrations ($P_{Birth}(K_\eta) = \xi[E]_\eta$), where ξ is the proliferation scale factor (Table S1). This results in a stem–like population of cells dependent on the underlying growth factor concentration. A cell (K_η at η) may divide into two identical daughter cells ($D_{1,2}$) if a randomly selected number $c_d(c_d \in U(0,1))$ is less than $\xi[E]_\eta$ regardless of there being an empty position. One daughter cell occupies the position of the parent cell (location of $D_1 = \eta_{x,y,z}$) while the other daughter cell (D_2) occupies one of five locations, being above or on one of the four orthogonal neighbors of the parent cell. This location is governed by ω, the division location probability, whereby the second daughter cell is placed in a position according to

$$\text{Location of } D_2(c_\omega, \omega) = \begin{cases} \eta_{x,y,z+h}, & if\ 0 \leq c_\omega \leq \omega, \\ \eta_{x+h,y,z}, & if\ \omega \leq c_\omega \leq \omega + \frac{1-\omega}{4}, \\ \eta_{x-h,y,z}, & if\ \omega + \frac{1-\omega}{4} \leq c_\omega \leq \omega + \frac{2(1-\omega)}{4}, \\ \eta_{x,y+h,z}, & if\ \omega + \frac{2(1-\omega)}{4} \leq c_\omega \leq \omega + \frac{3(1-\omega)}{4} \\ \eta_{x,y-h,z}, & if\ \omega + \frac{3(1-\omega)}{4} \leq c_\omega \leq 1, \end{cases}, \quad (3)$$

where η_{xyz} is the coordinates of the parent cell's lattice position, h is the grid size, and c_ω is a randomly selected number from (0,1) (i.e., $c_\omega \in U(0,1)$. Boundary conditions for cells are the same as those for our diffusible growth factor, being periodic on the left & right and front & back to enable spatial competition, no-flux on the bottom ($\eta_z = 0$), and Dirichlet (Cell $(\eta_x, \eta_y, Z) = 0$) on the top.

<u>NOTCH1 Advantage</u>

Upon induction of a mutation within NOTCH1, a fitness advantage is given to that cell and its progeny, if any. The fitness advantage gained through a NOTCH1 mutation is non-proliferative and allows the cell to remain within the basal layer longer than it would have otherwise. This is accomplished utilizing a blocking probability, f_o, which is independent for each cell regardless of clonal lineage. This indirectly impacts cell division at the basal layer, since

successful division requires displacement of neighboring cells towards the corneal layer. We assessed a range of f_o values up to $f_0 = 1.0$, where the tissue height is a quarter of its parameterized height.

TP53 Advantages

The total population size of a typical simulation over time for our homeostatic, spatial model can be defined by an ordinary differential equation model that describes population growth dynamics over time in a space limited condition. The equation is

$$\frac{dN}{dt} = rN\left(1 - \frac{N}{K}\right), \tag{9}$$

where N is the number of keratinocytes, r indicates a growth rate, K stands for a carrying capacity representing space limitation. Equation (9) allows us to approximate the same initializing conditions within the spatial model (3D-HCA) by initializing the model with the total number of cells in the simulated domain. Upon initialization the population reduces to the carrying capacity over time. In order to incorporate death from UV-damage we introduce a new component:

$$\frac{dN}{dt} = rN\left(1 - \frac{N}{K}\right) - \theta_s \mathbf{1}_s(t)N, \tag{10}$$

where the proportion of cells, θ_s, at time, t, is killed by sun exposure. The indicator function defined as,

$$\mathbf{1}_s(t) = \begin{cases} 1, & if\ t \in S, \\ 0, & otherwise. \end{cases} \tag{11}$$

The set of sun days, $S = \{t, t \in \mathbb{Z}, t \leq 365\}$ because $S \subset Y$ (the total days in a year), where UV damage occurs depends on the number of days chosen to repeat each year and the spacing of those days. We see that at realistic values for θ_s the ODE model is able to approximate our spatial simulations well, while a larger set of sun days and higher θ_s result in complete, or nearly complete, loss of tissue (Figure S7 and S8). In addition, the spatial model (3D-HCA) follows a logistic growth after a delayed period. This delayed period is exacerbated by higher values of θ_s and/or by less spacing in the number of sun days. This is the result of cells settling upon large death, creating a sparse tissue (in the absence of a TP53 mutant), the cells settle, partially, towards the basal layer prior to re-establishing homeostasis (Figure S7 and S8).

Here we assess a range of values for θ_s and sun day sets, S. The number of sun days varies for each individual as does the number of spacings between sun exposure days. We attempt to address both of these variables with regard to homeostasis. We assessed a number of sun days throughout the year (7, 20, and 100) and the spacing of those sun days (1 day to evenly spaced throughout the year). We can see that the fewer the number of sun days the least amount of tissue is lost (Figures S7 and S8). Likewise, the more spaced out these sun days are the closer to homeostasis the tissue remains (Figure S8). This is true for both the ODE and spatial models.

Model Parameterization

Parameter values for the spatial model were selected based on iterative redundancy analysis (RDA) using R(3) (vegan package(4)), where constraining variables are the emergent properties measured as a necessary component of homeostasis:

Primary Constraining Variables:

1. Mean Tissue height
2. Mean Loss/replacement rate within the basal layer

Secondary Constraining Variables:

1. Mean Overall tissue turnover time
2. Mean cell age
3. Mean Basal cell density

During the first iteration of parameterization variables are set randomly choosing values between zero and one for 20,000 two-year simulations. Diffusible parameters (ψ, γ, λ), where values were examined between zero and 1/6, were not subject to this iterative process. For the diffusion coefficient (ψ), values greater than 1/6 (for 3D, 0.25 in 2D) results in numerically unstable solutions for a given lattice point because it fails to satisfy Courant-Friedrichs-Lewy condition that guarantees convergence of a forward-time central-space finite difference method ($\psi \frac{\delta t}{h^2} \leq \frac{1}{6}$ (3D)). After each model run we collect the constraining variable data and in conjunction with the model parameters, we then perform RDA. Variance explained by each parameter are used to determine the influence of each parameter on the constraining variables. Refinement of the model parameters is then performed based on the variance explained and the linearity of that parameter on the constraining variables. In this way we are able to constrain the variable range iteratively by reducing the number of simulations to 5,000 (two-year simulations) while converging on parameter values. Iterations are repeated until target values are reached for each of the constraining variables of homeostasis where the combination of parameter values yields homeostasis. The secondary constraining variables served as a sanity check rather than a strict parameterization.

A pseudo-code for the parameterization process is as follows:

Function 1: Execute Simulations given parameter ranges:

For *n* simulations

1. Randomly select parameter values from provided ranges.
2. Run 2-year simulation of model.
3. Collect and calculate constraining variable values.

Function 2: RDA Analysis

While homeostasis is not reached:

1. Execute function 1 above (20,000 initially, 5,000 after).
2. Perform RDA and analyze parameter influence.
3. Further constrain ranges.
4. Repeat Step 1.

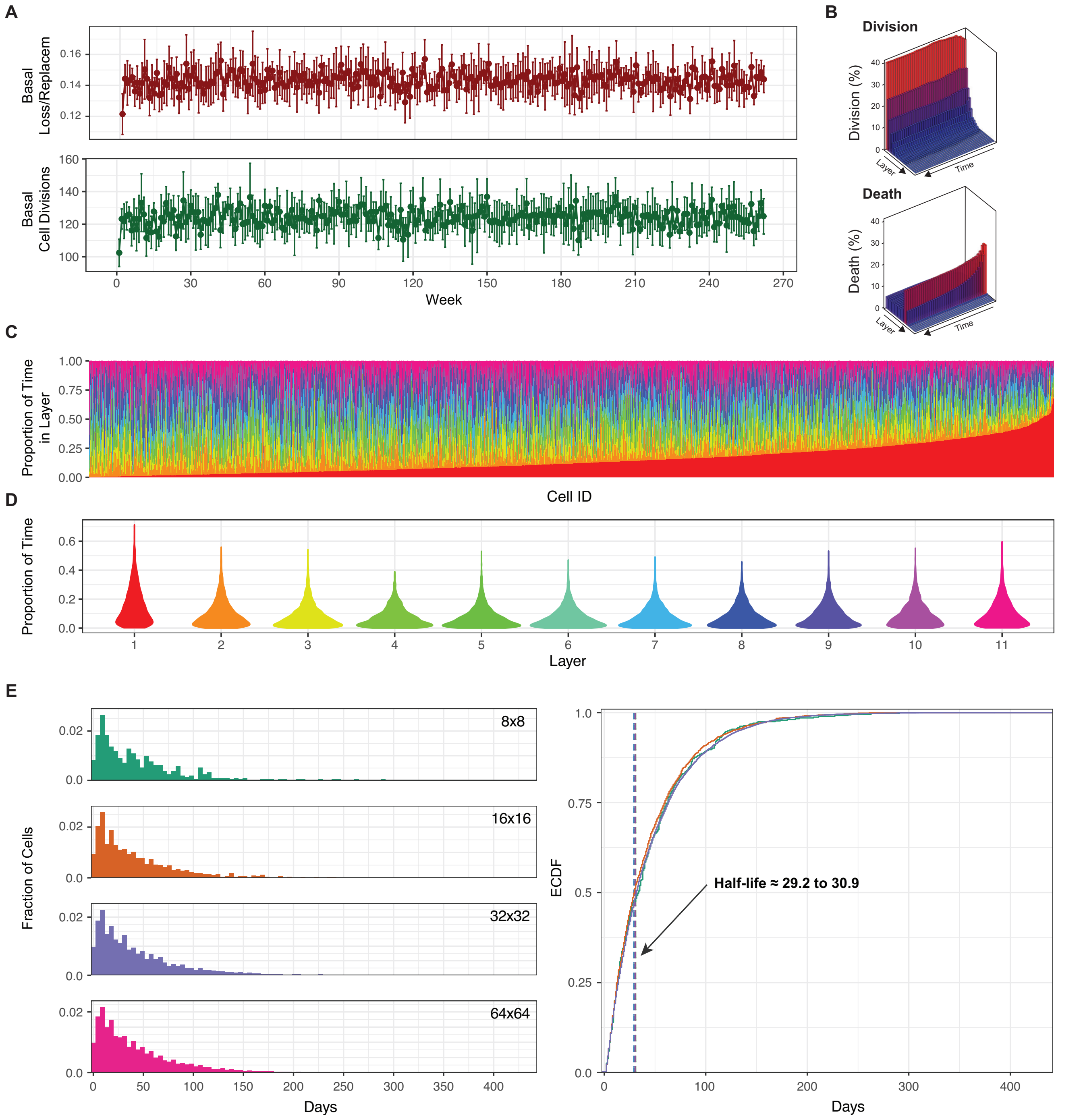

Fig. S1. Three dimensional hybrid cellular automata keratinocyte lifecycle. For a simulation of 1,024 cells (X and Y dimensions of 32x32) we see the weekly loss/replacement of the basal/progenitor cells (red) and the corresponding number of births (green)(**A**). For these same dimensions, the contributions of birth (top) and death (bottom) towards the overall population size of the simulated tissue are given for each layer of the epidermis over the course of a 2 year simulation (**B**). For **C** through **E** a three year period is simulated while positional and time information is only recorded for the second year. **C** and **D** show the proportion of time spent in each of the epidermis layers for each cell (**C**) or overall (**D**). **C** is sorted from least to greatest time spent in the basal layer for the time spent in the basal/progenitor layer and the colors for the layers are the same in **C** and **D**. **E** provides the distribution of time spent in the basal layer over four different domain sizes (**E**, left). The ECDF (**E**, right) distributions provide additional information for the half-life ($t_{1/2} = \tau ln(2)$, τ is the mean time in the basal/progenitor layer) of cells within the basal/progenitor layer.

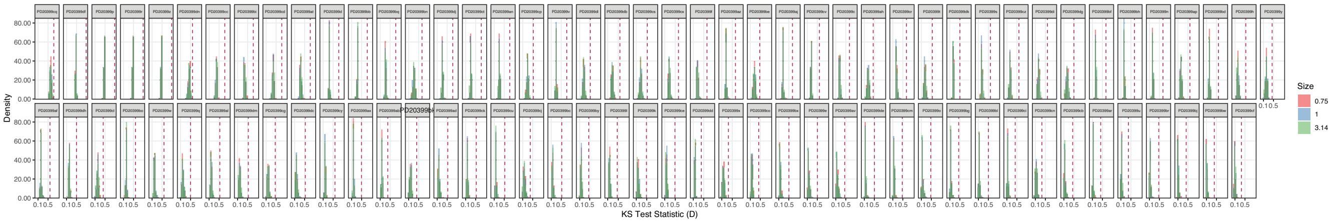

Fig. S2. Pairwise comparisons for 55 year old patient PD20399 illustrating the Komlogrov-Smirnov test statistic ($D_{m,n}$) distribution for the three biopsy simulation sizes. Minimum critical values (D_α) needed to reject the null hypothesis is illustrated by the red dashed line.

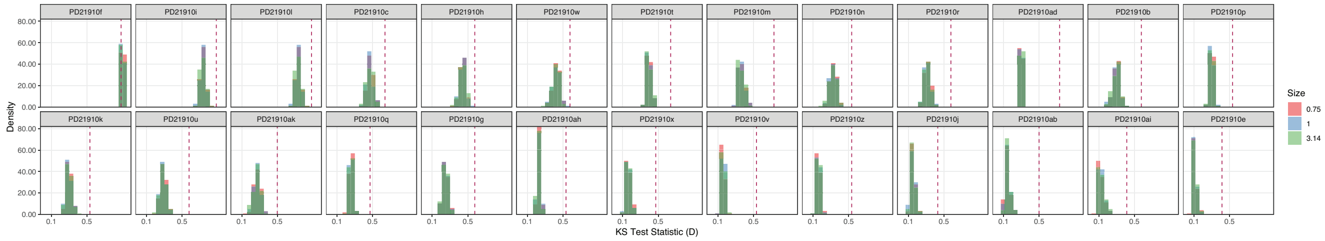

Fig. S3. Pairwise comparisons for 58 year old patient PD21910 illustrating the Komlogrov-Smirnov test statistic ($D_{m,n}$) distribution for the three biopsy simulation sizes. Minimum critical values (D_α) needed to reject the null hypothesis is illustrated by the red dashed line.

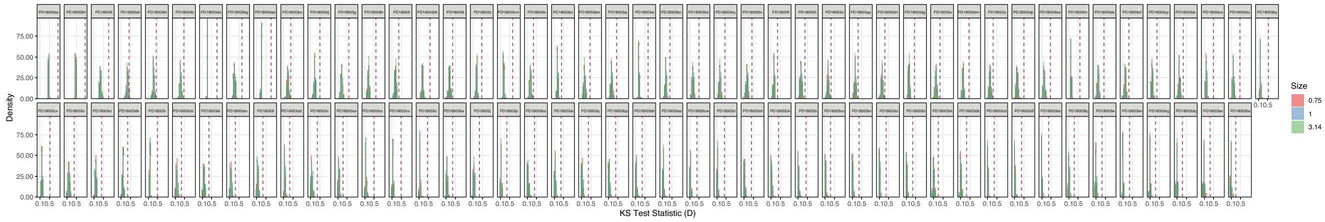

Fig. S4. Pairwise comparisons for 65 year old patient PD18003 illustrating the Komlogrov-Smirnov test statistic ($D_{m,n}$) distribution for the three biopsy simulation sizes. Minimum critical values (D_α) needed to reject the null hypothesis is illustrated by the red dashed line.

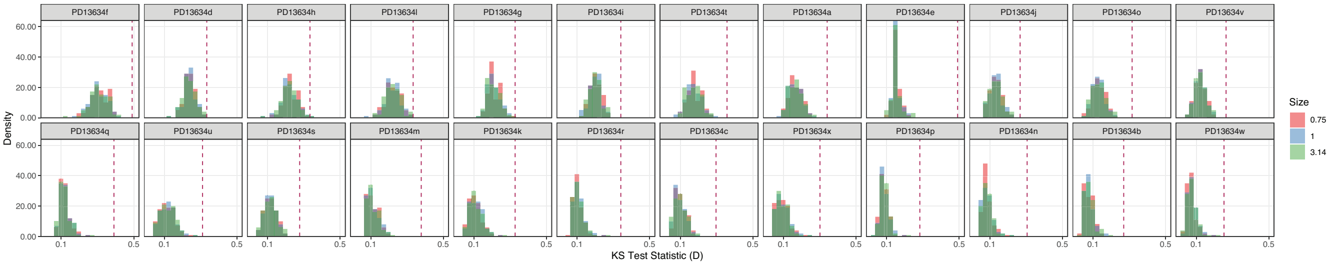

Fig. S5. Pairwise comparisons for 73 year old patient PD13634 illustrating the Komlogrov-Smirnov test statistic ($D_{m,n}$) distribution for the three biopsy simulation sizes. Minimum critical values (D_α) needed to reject the null hypothesis is illustrated by the red dashed line.

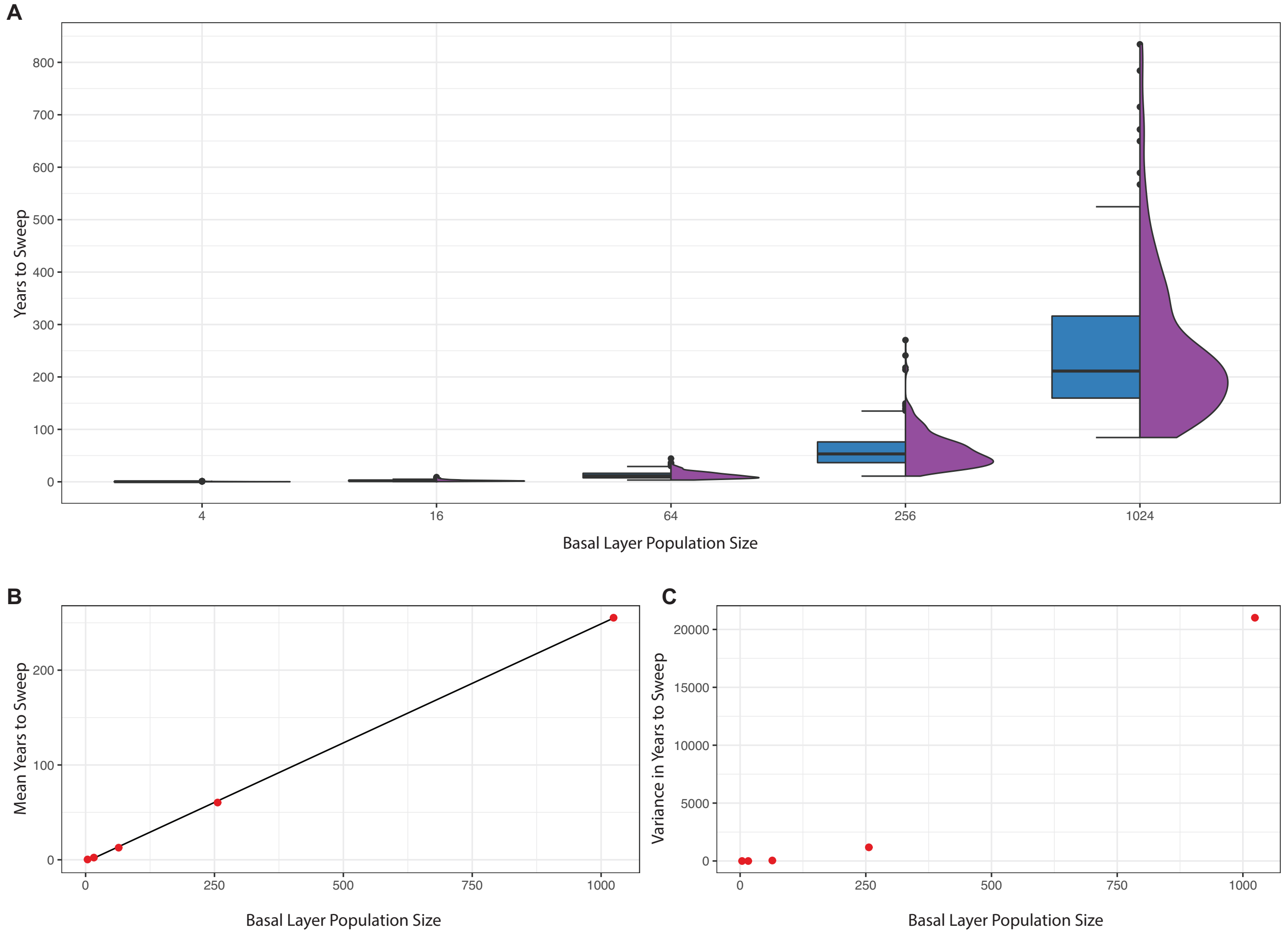

Fig. S6. Years for a single progenitor to sweep through basal layer. For 500 replicate simulations ranging from as small as 2x2 cells within the basal layer up to 32x32 cells (population size of 1024) within the basal layers we show the time it takes a single basal layer cell to sweep through the basal layer alone (**A**). For these same simulations a linear regression model ($r^2 = 0.9998$, p-value <0.001) is fit to the mean of the different simulations (**B**). While **B** illustrates the linearity of the mean time to sweep, the variance increases non-linearly as the population size increases (**C**).

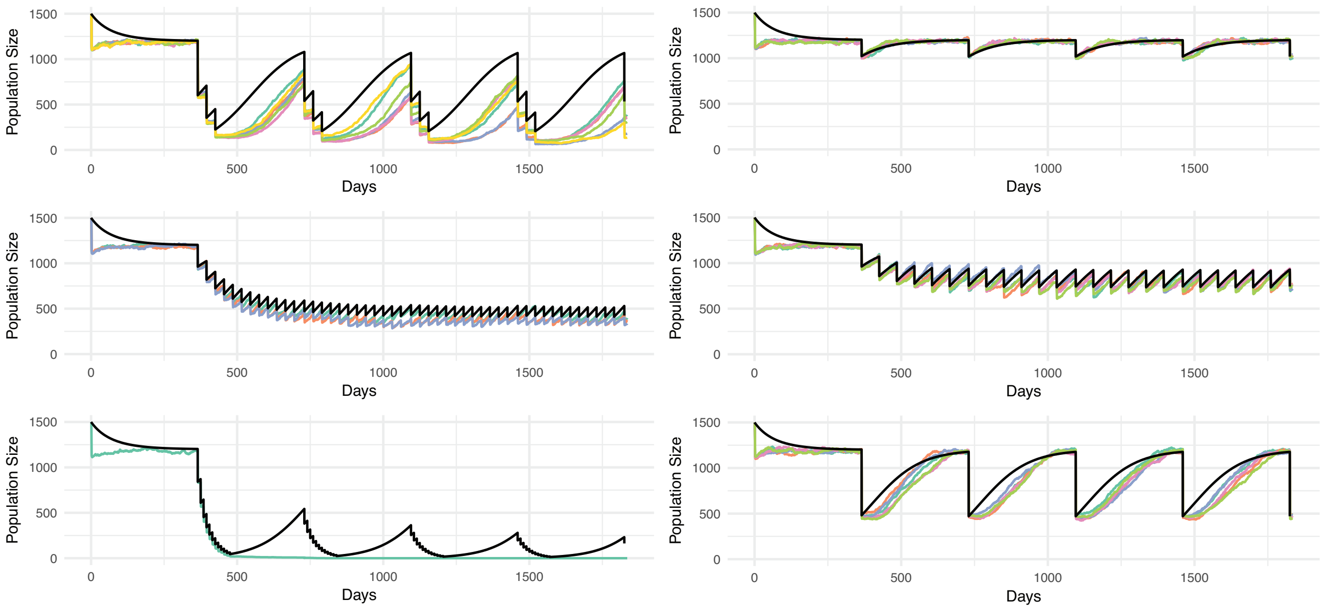

Fig. S7. Logistic growth model is able to capture spatial dynamics to aid in evaluating TP53 sun days and dynamics. Here we define various sun day spacings, S, and the proportion of cells killed during sun days, θ_s. Black line in each plot shows the ODE logistic growth model and the colored lines are for the spatial model (Methods). The first three plots on the left show severe and infrequent or, simply frequent perturbations. Whereas, for the right three plots, we see less severe perturbations through parameter combinations.

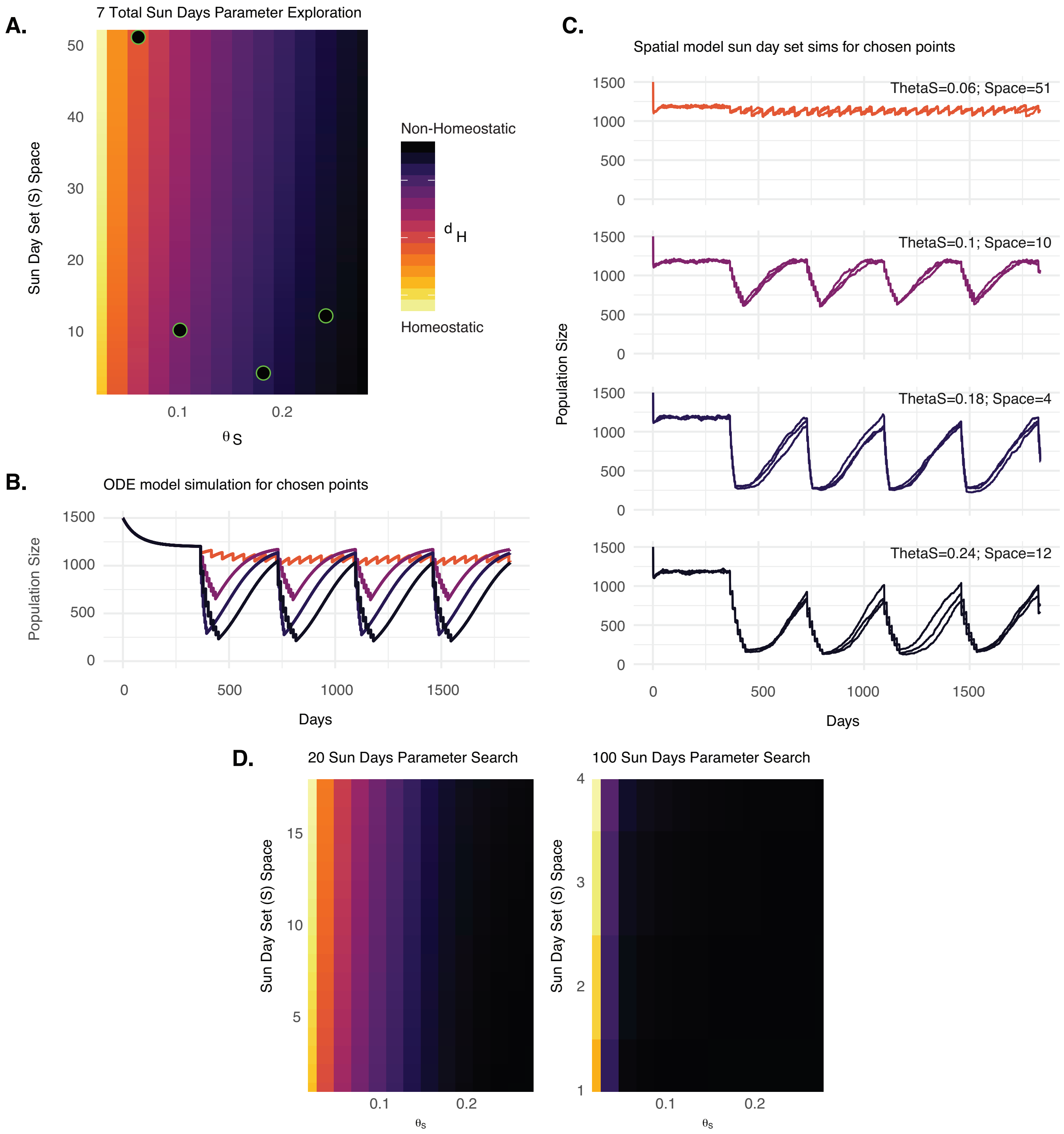

Fig. S8. TP53 parameter evaluation using a space limited ordinary differential equation (ODE). Evaluation of the number of sun days and θ_s values using the ODE model. In (**A**) we see the homeostatic measure d_H as a response to sun day spacing and θ_s repeating yearly. For each black point with a green border the population sizes over time is given for the ODE (**B**) and the spatial 3D-HCA model (**C**). For (**B**) and (**C**) the colored lines are colored for where they fall within (**A**). (**D**) shows the same information, but for different numbers of sun days. The available spacing of these sun days for (**A**) and (**D**) differ due to how many days are available to space the sun days throughout the year.

Parameter	Role
**3D-HCA Model Parameters**	
α	Level at which apoptosis can occur by chance.
θ	Random probability of death.
ξ	Scaling factor to control proliferation rate.
ϱ	Probability of moving into an empty position.
ω	Probability governing direction of division.
**Growth Factor Diffusible Parameters**	
**Parameter**	**Role**
E_c	Influx of GF at basal layer
ψ	Rate of GF diffusion from basal layer.
λ	Rate of GF decay.
γ	Rate of GF consumption by keratinocytes.

Table S1. Model Parameters. Parameters are separated into the cellular automata parameters and those belonging to the diffusible growth factor (GF).

Movie S1. Neutral Model Simulation (video S1.EpidermisNeutral.mp4). A $0.4mm^2$ 58 year neutral simulation. The initial and ending conditions are displayed on the upper corners of the panel. Each color represents a completely independent population (e.g. a population differing from its parent by at least one mutation). Time steps are shown and each frame represents a 6 month change.

References

1. E. Kim *et al.*, Senescent Fibroblasts in Melanoma Initiation and Progression: An Integrated Theoretical, Experimental, and Clinical Approach. *Cancer Research* **73**, 6874 (2013).
2. D. Basanta *et al.*, The Role of Transforming Growth Factor-β–Mediated Tumor-Stroma Interactions in Prostate Cancer Progression: An Integrative Approach. *Cancer Research* **69**, 7111-7120 (2009).
3. R. C. Team, R: A Language and Environment for Statistical Computing. (2018).
4. J. Oksanen *et al.*, vegan: Community Ecology Package. (2017).
